# Risk factors for postpartum maternal mortality and hospital readmission in low- and middle-income countries: a systematic review

**DOI:** 10.1186/s12884-023-05459-y

**Published:** 2023-04-29

**Authors:** Nicola E. Symonds, Marianne Vidler, Matthew O. Wiens, Shazmeen Omar, L. Lacey English, U. Vivian Ukah, J. Mark Ansermino, Joseph Ngonzi, Lisa M. Bebell, Bella Hwang, Astrid Christoffersen-Deb, Niranjan Kissoon, Beth A. Payne

**Affiliations:** 1 The Centre for International Child Health, BC Children’s Hospital, Vancouver, BC Canada; 2 Department of Obstetrics and Gynaecology, University of British Columbia, Rm V3-339, 950 West 28th Avenue, Vancouver, BC V5Z 4H4 Canada; 3 Department of Anesthesiology, Pharmacology and Therapeutics, Faculty of Medicine, University of British Columbia, Vancouver, Canada; 4 Faculty of Medicine, Mbarara University of Science and Technology, Mbarara, Uganda; 5 Walimu, Kampala, Uganda; 6 Department of Internal Medicine and Pediatrics, Hospital of the University of Pennsylvania, Philadelphia, PA USA; 7 Department of Epidemiology, Biostatistics and Occupational Health, McGill University, Montreal, QC Canada; 8 Department of Obstetrics and Gynaecology, Mbarara University of Science and Technology, Mbarara, Uganda; 9 Infectious Diseases Division, and Center for Global Health, Massachusetts General Hospital Medical Practice Evaluation Center, Boston, MA USA; 10 Department of Pediatrics, University of British Columbia, Vancouver, BC Canada; 11 School of Public and Population Health, University of British Columbia, Vancouver, BC Canada

**Keywords:** Postpartum period, Maternal mortality, Readmission, Post-discharge risk, Low- and middle-income countries

## Abstract

**Background:**

In low- and middle-income countries, approximately two thirds of maternal deaths occur in the postpartum period. Yet, care for women beyond 24 h after discharge is limited. The objective of this systematic review is to summarize current evidence on socio-demographic and clinical risk factors for (1) postpartum mortality and (2) postpartum hospital readmission.

**Methods:**

A combination of keywords and subject headings (i.e. MeSH terms) for postpartum maternal mortality or readmission were searched. Articles published up to January 9, 2021 were identified in MEDLINE, EMBASE, and CINAHL databases, without language restrictions. Studies reporting socio-demographic or clinical risk factors for postpartum mortality or readmission within six weeks of delivery among women who delivered a livebirth in a low- or middle-income country were included.

Data were extracted independently by two reviewers based on study characteristics, population, and outcomes. Included studies were assessed for quality and risk of bias using the Downs and Black checklist for ratings of randomized and non-randomized studies.

**Results:**

Of 8783 abstracts screened, seven studies were included (total *N* = 387,786). Risk factors for postpartum mortality included Caesarean mode of delivery, nulliparity, low or very low birthweight, and shock upon admission. Risk factors for postpartum readmission included Caesarean mode of delivery, HIV positive serostatus, and abnormal body temperature.

**Conclusions:**

Few studies reported individual socio-demographic or clinical risk factors for mortality or readmission after delivery in low- and middle-income countries; only Caesarean delivery was consistently reported. Further research is needed to identify factors that put women at greatest risk of post-discharge complications and mortality. Understanding post-discharge risk would facilitate targeted postpartum care and reduce adverse outcomes in women after delivery.

**Trial registration:**

PROSPERO registration number: CRD42018103955.

**Supplementary Information:**

The online version contains supplementary material available at 10.1186/s12884-023-05459-y.

## Background

The proportion of maternal deaths occurring in the postpartum period is increasing globally [1–3]. In low- and middle-income countries (LMICs), approximately two-thirds of maternal deaths occur in the postpartum period. Despite more women delivering in health facilities, which is known to improve outcomes due to appropriate and timely access to skilled care [4, 5], many women are still at high risk of death [6]. Among women who die in the postpartum period, 80% of deaths occur within one week of delivery [7]. Women who do deliver in health facilities are typically discharged within 24 h without further follow-up, resulting in a gap in care during a high-risk period [6]. Further, postpartum care beyond 24 h after delivery is often limited, evidenced by the fact that the majority of women do not complete the three recommended postnatal visits [8–10].

Studies in high-income countries (HICs) have begun to evaluate risk factors for adverse postpartum and post-discharge outcomes [11, 12], yet little is known regarding the risk factors for complications or readmission following delivery in LMICs where postpartum morbidity and mortality is greatest. Stillbirth has previously been reported in the literature as a known risk factor for postpartum mortality and is responsible for a disproportionate number of deaths after pregnancy compared to a livebirth [13, 14]. However, to our knowledge, few studies have identified risk factors other than stillbirth for postpartum mortality or readmission in LMICs. The ability to identify women at higher risk of postpartum complications prior to discharge could help reduce mortality and morbidty. An improved understanding of how to estimate postpartum risk is essential for designing and implementing targeted interventions in the postpartum period.

The objective of this systematic review is to summarize current evidence on individual socio-demographic and clinical risk factors for (1) postpartum mortality and (2) postpartum hospital readmission that could be used for post-discharge risk stratification in LMICs. The ultimate goal of this review is to identify individual risk factors that could be used to determine if a women is at high risk after delivery for postpartum death or complication requiring readmission to hospital. We aim to compel further research and eventually develop interventions to reduce maternal mortality and rehospitalization in the postpartum period.

## Methods

### Search strategy

This systematic review was conducted according to PRISMA guidelines [15] and was registered on PROSPERO prior to completing the final search (CRD4201813955). Articles published from database inception to January 9, 2021 were identified using MEDLINE, EMBASE, and CINAHL databases, with no language restrictions. References of systematic reviews and included studies were also reviewed. Search terms included a combination of keywords and subject headings (i.e. MeSH terms). See online supplement for each database’s full search strategy (see Additional files 1, 2 and 3). The search was conducted by the first author with input from co-authors and a research librarian. Covidence was used to manage citations throughout the review process [16].

### Study selection

Titles and abstracts were independently screened by two reviewers to determine eligibility. Conflicts were resolved by a third reviewer with expertise in obstetrics. Two authors then assessed all full-text articles for inclusion. Studies examining individual socio-demographic and clinical risk factors for postpartum mortality or hospital readmission (within six weeks) among women delivering in a low- or middle- income country were included. Studies that did not differentiate between ante-, intra- and/or post-partum outcomes were not included. We excluded papers looking exclusively at outcomes following abortions, stillbirths, or home deliveries. See Table 1 for details on inclusion criteria.

**Table 1:** PICOS Inclusion Criteria

**Population**	Postpartum women who delivered in a LMIC, as defined by those countries currently (2019) classified by the UNDP^a^ as having low to middle HDI^b^ [17] Exclusions: • No maternal data or maternal data not well differentiated from other populations • No disaggregation between ante-, intra- and post-partum results • Maternal data exclusively following abortions • Maternal data exclusively following stillbirths • Maternal data exclusively following home deliveries
**Interventions**	Usual care, or care during an intervention of any kind
**Comparisons**	None
**Outcomes**	***Primary outcomes:*** • Risk factors for postpartum* maternal mortality • Risk factors for postpartum* maternal readmission Risk factors include location and mode of delivery, and socio-demographic or clinical characteristics. Risk factors must be identified and evaluated with reported measures of association, such as odds ratio or risk ratio, or provide necessary data to calculate a measure of association ***Secondary outcomes:*** • Postpartum maternal mortality rate • Postpartum maternal readmission rate *Postpartum period defined using the WHO^c^ standard of within six weeks of delivery [18]
**Study Design**	Eligible study designs include the following: • Randomized control trials • Prospective or retrospective cohort studies • Case–control studies • Studies utilizing surveillance data Exclusions: • Case reports • Commentaries • Conference abstracts • Review articles (including systematic reviews)

^a^United Nations Development Programme; ^b^ Human Development Index; ^c^ World Health Organization

### Data extraction and quality assessment

Data were extracted in Microsoft Excel based on study characteristics, population and outcomes, using odds ratio as the primary measure of association for all identified risk factors (Table 2). Two authors each extracted data for half the eligible studies and reviewed the other half for accuracy and consistency. If risk factors and their respective measures of association were not directly reported, the odds ratio was independently calculated. Included studies were assessed for quality and risk of bias using the Downs and Black checklist for randomized and non-randomized studies (see Additional file 4) [19]. Modifications to this checklist were made to allow for all included studies to be assessed. Specifically, the quality item for reporting adverse events related to the intervention was removed and the final question on power was changed to a yes/no response. ‘Yes’ was chosen if the study reported a sample size or power calculation representative of the main study aim and design.

**Table 2:** Study characteristics

**Author**	**Study country**	**Study period**	**Study design**	**Population (sample size)**	**Risk factor**	**Risk factors for postpartum mortality**	**Risk factors for postpartum readmission**	**Postpartum mortality ratio (rate per 100,000 deliveries)**	**Postpartum readmission rate (%)**
Bebell [20]	Uganda	Mar-Oct 2015	Prospective cohort	General (*n* = 1785)	HIV^a^ serostatus	N	Y	Not applicable	1.7%
Harrison [21]	Guatemala, India, Kenya, Pakistan, Zambia and the DRC^b^	Jan 2010-Dec 2015^c^	Prospective cohort	General (*n* = 384,461)	Mode of delivery	Y	Y	100	3.9%
Kanyighe [13]	Malawi	2001–2002	Retrospective case–control	General (*n* = 209)	Mode of delivery, parity, birthweight, Apgar score	Y	N	Not reported	Not applicable
Ngonzi [22]	Uganda	Not reported	Prospective cohort	General (*n* = 1913)	Body temperature	N	Y	Not applicable	1.5%
Oladapo [23]	Nigeria	Jan 1990-Dec 2005	Retrospective descriptive	General (*n* = 820)	Mode of delivery	Y	Y	400	2.0%
Igbaruma [24]	Nigeria	Jan 2009-Dec 2012	Prospective observational	ICU admissions (*n* = 95)	Age, marital status, referral status, education, place of delivery	Y	N	Not reported	Not applicable
Ojengbede [25]	Nigeria	Mar 2004-Jan 2008	Pre/post-intervention	Postpartum hemorrhage (*n* = 288)	Severity of shock, parity	Y	N	10,800	Not applicable

^a^ Human Immunodeficiency Virus; ^b^ Democratic Republic of the Congo; ^c^ DRC initiated enrollment in 2014

### Data synthesis

The primary outcomes in this review were risk factors for postpartum mortality or readmission. Risk factors were defined as individual socio-demographic or clinical characteristics, including characteristics of the woman giving birth (e.g. age, parity, medical history, symptoms and laboratory test results) or the care she received during delivery (e.g. location or mode of delivery). The secondary outcomes evaluated were rates of postpartum mortality or readmission (Table 2). A meta-analysis was not conducted due to variation among the study population, risk factors, and analysis. Studies were organized by population type (i.e. general obstetric population, postpartum hemorrhage, intensive care unit (ICU) admissions), and types of risk factors assessed (Tables 3 and 4).

**Table 3:** Risk factors for postpartum mortality

Risk factor	Included study	Odds ratio (95% CI)	Risk factor definition	Reference comparator
***General maternal population***				
Mode of delivery	Harrison et al., 2017 [21]	2.42 (1.95–3.01)	CS^a^	VD^b^ (includes both spontaneous and assisted vaginal deliveries)
	Kanyighe et al., 2008 [13]	7.38 (2.08–26.07)	CS	CS not conducted
	Oladapo et al., 2007 [23]	2.01 (0.03–38.83)	Elective CS	Non-operative VD following spontaneous onset of labour
Parity	Kanyighe et al., 2008 [13]	3.34 (1.46–7.66); 1.37 (0.50–3.72)	Nulliparous mothers; high parity (> 3 previous births)	Low parity (1–3 previous births)
Birthweight	Kanyighe et al., 2008 [13]	83.23 (10.24–676.2); 3.51 (1.85–6.63)	Very low birth weight (< 1.5 kg); low birth weight (1.5 kg-2.5 kg)	Normal birth weight (> 2.5 kg)
Apgar score	Kanyighe et al., 2008 [13]	1.67 (0.31–8.94)	Low score (1–5)	Normal score (6–10)
***ICU admissions population***				
Age	Igbaruma et al., 2016 [24]	0.44 (0.18–1.11)	Mothers < 20 & ≥ 35 years	Mothers aged 20–34 years
Marital status	Igbaruma et al., 2016 [24]	0.73 (0.26–7.02)	Single	Married
Referral status	Igbaruma et al., 2016 [24]	0.98 (0.40–2.40)	Not referred	Referred
Education	Igbaruma et al., 2016 [24]	1.39 (0.49–3.91)	Secondary maternal education or less	Post-secondary maternal education
Place of delivery	Igbaruma et al., 2016 [24]	0.44 (0.10–1.97); 0.30 (0.04–2.20)	Primary health facility; home/informal maternal setting	Delivery in a secondary/tertiary facility
***Postpartum hemorrhage population***				
Severity of shock upon admission	Ojengbede et al., 2011 [25]	6.84 (1.55–30.19)	MAP^e^ < 60	MAP ≥ 60
Parity	Ojengbede et al., 2011 [25]	0.91 (0.40–2.06)	High parity (5 or more)	Low parity (0–4)

^a^ Caesarean Section; ^b^ Vaginal Delivery; ^c^ 23 300 IU of preformed vitamin A as retinyl palmitate; ^d^ 42 mg of all *trans* ß carotene; ^e^ Mean Arterial Pressure

**Table 4:** Risk factors for postpartum readmission

Risk factor	Included study	Odds ratio (95% CI)	Risk factor definition	Reference comparator
***General maternal population***				
HIV^a^ status	Bebell et al., 2018 [20]	2.60 (1.15–5.89)	HIV-infected	HIV-uninfected
Mode of delivery	Harrison et al., 2017 [21]	5.15 (4.97–5.33)	CS^b^	VD^c^ (includes both spontaneous and assisted vaginal deliveries)
	Oladapo et al., 2007 [23]	3.21 (1.06–9.56)	Elective CS among booked patients	Non-operative VD following spontaneous onset of labour
Body temperature during hospitalization	Ngonzi et al., 2018 [22]	12.78 (6.01–27.17)	Febrile (> 38.0 °C) or hypothermic (< 36.0 °C) women	Normothermic women

^a^ Human Immunodeficiency Virus; ^b^ Caesarean Section; ^c^ Vaginal Delivery

## Results

**Fig. 1:**
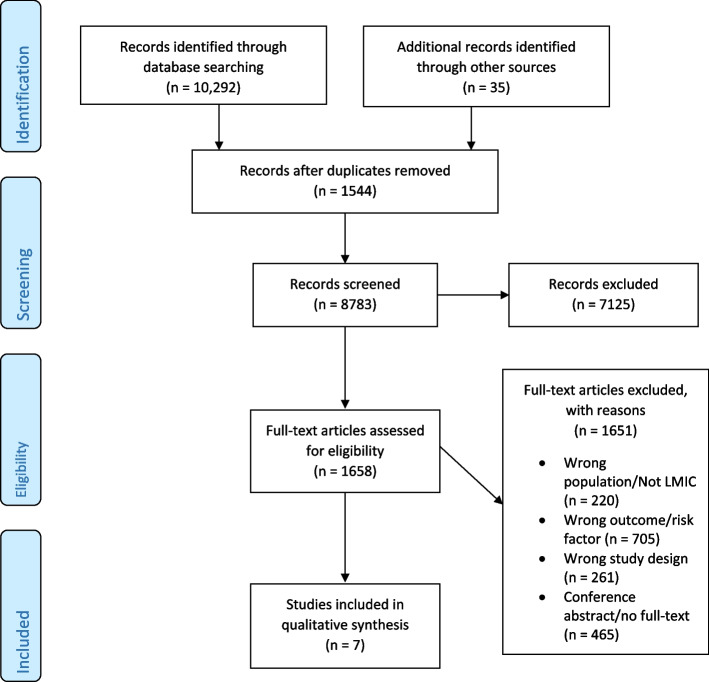
PRISMA Flow Diagram

A total of 8783 abstracts were screened. Of these, 7125 were excluded after abstract and title screening, leaving 1658 full-text manuscripts for review. After full-text screening was completed, seven studies were included (Fig. 1) [13, 20–25].

Study characteristics can be found in Table 2. Publication dates ranged from 2007 to 2018, four out of seven (57%) were published after 2015. Study designs included four prospective cohort studies, one pre/post-intervention study, and two retrospective studies. Most studies (six out of seven) were conducted in sub-Saharan Africa. One study contained data from six countries [21], with three sites in sub-Saharan Africa and four across India, Pakistan and Guatemala. The aim of this present review was to assess post-discharge risk among facility deliveries, however, two included studies used a combination of facility and home deliveries [21, 24] and three did not specify delivery location [20, 22, 25].

Overall, the risk of bias in the included studies was moderate to low with some issues identified in the quality of reporting and internal validity. The included studies ranged in score from 14 to 22 out of a total of 26 points on the modified Downs and Black scale (see Additional file 4).

Five studies focused on a general maternal population, while the remaining two studies targeted sub-populations: those diagnosed with and admitted for postpartum hemorrhage (PPH) (750 mL of blood loss or greater) after delivery [25], or those with an obstetric admission to the ICU anytime between 24 weeks gestation up to six weeks of delivery [24]. For the purpose of this review, only data among postpartum ICU admissions were reported. Postpartum mortality and readmission rates were reported as secondary outcomes (Table 2). The overall postpartum mortality ratio ranged from 100 to 10,800 deaths per 100,000 deliveries in the included studies. The postpartum readmission rate reported in included studies ranged from 1.5 to 3.9%. Rates were either reported directly from the study or calculated by the authors where data was available. Four studies reported a measure of association for risk factors [13, 23–25], while the odds ratio was calculated using 2 × 2 tables for the remaining three studies [20–22].

### Risk factors for postpartum mortality

Five studies enrolling between 95 and 384,461 women per study reported risk factors for postpartum mortality (Table 3) [13, 21, 23–25]. Three of these studies evaluated risk factors among a general maternal population [13, 21, 23], and the fourth and fifth study evaluated risk factors among a maternal population admitted to the ICU [24] and a population of women with postpartum hemorrhage [25], respectively.

Among the three studies evaluating a general maternal population, risk factors for postpartum mortality included delivery by Caesarean section (CS) [13, 21, 23], high parity or nulliparity [13], low or very low birthweight [13], and an Apgar score of 5 or less [13]. Of these, CS delivery, nulliparity, and low or very low birthweight were statistically significant.

Mode of delivery was most frequently reported in relation to postpartum mortality; however, variation in how it was defined did not permit further grouped analysis. All three studies found delivery by CS was associated with an increased risk of postpartum mortality, although one study was not statistically significant, with the odds of mortality ranging from 2.01 (95% CI: 0.03–38.83) to 7.38 (95% CI: 2.08–26.07). These three studies included both elective and emergency CS. One study evaluated the risk of elective CS compared to non-operative vaginal delivery (VD) following spontaneous onset of labour [23]. Another evaluated the risk of any CS compared to any VD, including assisted and spontaneous deliveries [21]. The third study evaluated the risk of postpartum mortality among women who underwent CS compared to those who did not, with no further characteristics describing the mode of delivery [13].

Aside from mode of delivery, parity, infant birthweight and Apgar scores were also assessed [13]. Risk factors found to be significant were nulliparity (OR: 3.34, 95% CI: 1.46–7.66), low birthweight between 1.5 and 2.5 kg (OR: 3.51, 95% CI: 1.85–6.63), and very low birthweight below 1.5 kg (OR: 83.23, 95% CI: 10.24–676.2). Low Apgar scores (between 1 and 5) and high parity (more than 3 previous births) were also assessed, but neither were statistically significant.

Among the maternal population admitted to the ICU within six weeks of delivery, none of the reported risk factors for postpartum mortality were statistically significant [24]. The non-significant risk factors identified were maternal age of 20 to 34 years, married women, referred women, women delivering at a secondary or tertiary health facility, and women with a secondary level education or below (Table 3).

Among women with postpartum hemorrhage, risk factors for postpartum mortality included severe shock upon admission and high parity, with the former being statistically significant [25]. Specifically, severe shock, defined as a mean arterial pressure (MAP) below 60 or a non-palpable blood pressure, upon admission predicted a six-fold increase in the odds of postpartum mortality compared to women with a MAP of 60 or above (OR: 6.84, 95% CI: 1.55–30.19). High parity, defined as five or more livebirths, was associated with a reduced likelihood of postpartum mortality, but this finding was not statistically significant.

### Risk factors for postpartum readmission

Four studies enrolling 820 to 384,461 women per study reported risk factors for postpartum readmission (Table 4) [20–23]. Three of these studies described hospital readmissions occurring within six weeks of delivery [20–22], while one study evaluated readmission for problems related to mode of delivery but did not specify the time period [23]. All reported risk factors for readmission were statistically significant, including CS delivery [20, 22], human immunodeficiency virus (HIV) positive serostatus [20], and abnormal body temperature during hospitalization [22]. Similar to mortality, mode of delivery was the most commonly reported predictor of postpartum readmission. Both studies found delivery by CS was associated with an increased risk of readmission compared to VD; however, nuances exist within this category of CS delivery. Odds of readmission were three times higher (OR: 3.21, 95% CI: 1.06–9.56) among those who had an elective CS [23] and five times higher (OR: 5.15, 95% CI: 4.97–5.33) among those with any CS delivery [21]. Bebell et al. [20] found HIV seropositive women were significantly more likely to be readmitted to hospital in the postpartum period compared to HIV seronegative women (OR: 2.60, 95% CI: 1.15–5.89). Body temperature abnormalities showed the largest risk estimate for postpartum readmission. Women who were febrile (> 38.0 °C) or hypothermic (< 36.0 °C) during their hospital stay were nearly thirteen times more likely (OR: 12.78, 95% CI: 6.01–27.17) to be readmitted in the postpartum period than normothermic women [22].

## Discussion

This systematic review of risk factors for maternal death or readmission after delivery in LMICs identified only seven eligible studies, mostly from sub-Saharan Africa. We identified four significant risk factors for maternal mortality in the postpartum period, specifically CS delivery, nulliparity, low or very low birthweight, and severe shock upon admission. Three significant risk factors for postpartum readmission were identified, notably CS delivery, HIV positive serostatus, and abnormal body temperature.

CS delivery was a commonly reported risk factor associated with an increased risk of both postpartum mortality and readmission. This fits with the existing literature demonstrating that CS increases the risk for postpartum complications, such as postpartum sepsis [26–28]; however, these women may already be high-risk due to underlying conditions or complications [29]. In this context, CS acts as a proxy, representing risk related to other underlying conditions. For this study, we are not concerned with the causal pathway between these underlying conditions and mortality, so CS is still valuable as a candidate predictor for further research. Research has also found emergency CS to be associated with a higher risk of poor maternal outcomes than elective CS [30, 31]. Despite several studies in this review reporting increased risk of postpartum death or readmission related to CS, the heterogeneity in the definitions used and the lack of information regarding indication for CS did not allow for a meta-analysis. To permit more consistent reporting, analysis, and comparison of CS outcomes, the use of a standardized, global definition for CS such as the Robson classification is recommended in future studies [32]. Findings from this review support the WHO recommendations for postpartum follow-up within 24 h, between 24–72 h, between 7 and 14 days, and six weeks postpartum. Febrile or hypothermic women during hospitalization in Uganda were nearly thirteen times more likely to be readmitted in the postpartum period compared to normothermic women. Temperature has also been found to be a risk factor for other adverse outcomes, including maternal mortality and post-discharge infant mortality [33, 34]. Kanyinghe et al. [13] also reported an increase risk of death in women with postpartum infection, but the method of diagnosing infection was not provided in the paper, making interpretation of this finding difficult and thus was not included in the results above. This review highlights the need for clear definitions of risk factors for generalizability, study replication, and meta-analysis.

This systematic review has several strengths. Firstly, the search strategy was broad to ensure all relevant studies would be captured. Despite the thousands of citations screened, less than 0.1% were included. There was a significant number of studies that failed to meet inclusion criteria for this review because they did not disaggregate timing of maternal death when reporting results. This is a testament to the distinct lack of evidence surrounding this topic, which merits further investigation. We recommend that the global maternal health research community adopt the practice of disaggregating results by timing on maternal death and severe morbidity. It is clear that the causes of maternal death differ by timing [3] and it is likely the risk factors associated with each of these time periods and related causes would also differ. In order to improve care and reduce deaths and severe morbidities in each of the antepartum, intrapartum and postpartum period, data specific to each time period is needed. In addition, a rigorous review process was used with at least two independent reviewers assessing each study.

This review also has several limitations. Firstly, the intention was to capture data for post-discharge maternal mortality and readmission after facility delivery. Given the paucity of data targeting post-discharge maternal outcomes, any outcomes referred to as “postpartum” were included. These postpartum outcomes were used as a proxy for post-discharge risk, yet the postpartum period could have been defined differently and not specified in some cases. Studies lacking a clear definition of the postpartum period may have included intrapartum deaths, or outcomes beyond six weeks. Some studies did not define the follow-up period and merely referred to outcomes occurring within the postpartum period; therefore, we assumed use of the World Health Organization definition of a six-week postpartum period [18]. Similarly, the definition of a hospital readmission may vary. Studies may have been limited to measuring readmissions occurring at the same hospital as the delivery, which could have resulted in an underestimation of the true number of readmissions. For example, some patients may have been readmitted to other hospitals or local health centers, but this data was not necessarily available or reported. In addition, some studies only included data during admission which may underrepresent the true outcome rates and result in missing risk factors. Secondly, two studies were both secondary analyses of the same dataset [20, 22]. However, because each study reported different risk factors for postpartum readmission (i.e. HIV-positive serostatus and abnormal body temperature) and a meta-analysis was not conducted, we included the data from both studies. We are aware there may be overlap between these two populations and this may have been the case for other studies taking place in the same region. While three of the seven included studies took place in Nigeria, we think it is unlikely the same data and populations were used given these studies all differed by time period of data collection and study design. Thirdly, we excluded studies looking exclusively at outcomes following only abortions or stillbirths due to the inherent difference in risk for adverse events among these populations compared to women who delivered livebirths. However, some post-abortion or post-miscarriage data may have been included because these outcomes were not always specified by the included studies. Nevertheless, it cannot be ignored that stillbirth is a risk factor for postpartum mortality [13, 14]. Existing literature suggests low socioeconomic status, lack of antenatal care, history of stillbirth, and poor maternal nutrition increase risk of stillbirth [35–37]. It is reasonable to assume these factors would also predict post-discharge maternal mortality or readmission. This warrants further investigation.

Despite the increasing number of facility deliveries in LMICs and increasing rate of postpartum mortality, very little research has examined post-discharge outcomes [6, 38]. To our knowledge, this is the first systematic review of individual socio-demographic or clinical factors that predict mortality or readmission after delivery in LMICs. We chose to focus on individual and measurable socio-demographic and clinical risk factors, such as parity and mode of delivery, as these can be applied consistently across geographies to identify risk. Research on predictors of risk in the postpartum period must be independently validated within individual regions prior to any clinical use.

There is a clear gap in understanding which factors may contribute to poor outcomes after delivery and discharge from facility in low-resource settings. However, exploration of these issues has begun to emerge in HICs, such as the United States-based analysis of postpartum readmission [11]. This study examined hospital readmissions within six weeks of delivery. CS delivery and maternal comorbidities, including hypertension and psychiatric disease, were found to be strong predictors for postpartum readmission [11].

This systematic review explored risk factors for postpartum mortality and readmission; however, there is a critical need for further research on this topic. This review highlights a gap in understanding predictors of adverse post-discharge maternal outcomes, as evidenced by the small number of included studies. Post-discharge maternal mortality and readmissions data in LMICs is rarely presented. A small number of studies in HICs demonstrate this data is feasible to collect and relevant to report [11, 12]. Establishing a set of socio-demographic and clinical characteristics with predictive power for determining death or readmission after delivery would have wide-ranging implications. Understanding what puts some women at higher risk after facility delivery could facilitate the design of targeted postpartum care. Creating differentiated pathways of postpartum or post-discharge care based on reliable risk stratification could improve health outcomes and economic efficiency of health systems. Studies are currently underway, including the Smart Discharges program in Uganda, to address this gap for infants and children [39, 40]. Investigation of post-discharge outcomes in LMICs has begun to develop in pediatric research [33, 41], and a similar focus should be applied within the field of maternal health. This is especially important in low-resources settings where the health burden is the greatest and distribution of scarce resources must be optimized. We recognize that a one-size fits all approach is not valuable and recommend that research in this area be targeted to specific individual countries to allow for the influence of varying health system structures, and social and economic determinants of health on maternal risk. By identifying women most at-risk following delivery, we can target interventions for these women, increase health resource efficiency and, most importantly, reduce mortality and complications.

## Conclusion

We have identified few risk factors for postpartum mortality and readmission in LMICs from the existing literature; the most common was delivery by cesarean section. Identification of this significant gap in the literature should serve as a call to action for further research to determine what increases the risk of post-discharge complications and mortality for women after delivery. Any research on maternal death or severe morbidity should report data disaggregated by timing of outcome to allow for improvements in postpartum care. There is an important opportunity to tailor recommendations for timing and frequency of follow-up and to guide the use of scarce healthcare resources to intervene with those at highest risk. The development of an evidence base upon which to build recommendations for the timing and intensity of postnatal care in resource-poor countries based on individualized risk prediction is urgently needed.

## Supplementary Information

**Additional file 1.** MEDLINE search strategy (database inception – January 9, 2021).

**Additional file 2.** EMBASE search strategy (database inception – January 9, 2021).

**Additional file 3.** CINAHL search strategy (database inception – January 9, 2021).

**Additional file 4.** Downs and Black checklist for the assessment of the methodological quality of both randomized and non-randomized studies.

**Additional file 5.**

## Data Availability

The datasets used during the this study are included in this published article and available under supplementary materials.

## References

[1] Kassebaum NJ, Bertozzi-Villa A, Coggeshall MS (2014). Global, regional, and national levels and causes of maternal mortality during 1990–2013: a systematic analysis for the Global Burden of Disease Study 2013. Lancet.

[2] Say L, Chou D, Gemmill A, Tunçalp Ö, Moller A-B, Daniels J, Gülmezoglu AM, Temmerman M, Alkema L (2014). Global causes of maternal death: a WHO systematic analysis. Lancet Glob Health.

[3] Merdad L, Ali MM (2018). Timing of maternal death: Levels, trends, and ecological correlates using sibling data from 34 sub-Saharan African countries. PLoS ONE.

[4] Nieburg P. Improving Maternal Mortality and Other Aspects of Women’s Health. 2012. https://www.csis.org/analysis/improving-maternal-mortality-and-other-aspects-women%E2%80%99s-health. Accessed 8 Mar 2022

[5] World Health Organization, Midwives IC of, d’Obstétrique F internationale de G et. Making pregnancy safer : the critical role of the skilled attendant : a joint statement by WHO, ICM and FIGO. World Health Organization. 2004.

[6] Ahmed I, Ali SM, Amenga-Etego S (2018). Population-based rates, timing, and causes of maternal deaths, stillbirths, and neonatal deaths in south Asia and sub-Saharan Africa: a multi-country prospective cohort study. Lancet Glob Health.

[7] Li XF, Fortney JA, Kotelchuck M, Glover LH (1996). The postpartum period: the key to maternal mortality. Int J Gynaecol Obstet.

[8] Langlois ÉV, Miszkurka M, Zunzunegui MV, Ghaffar A, Ziegler D, Karp I (2015). Inequities in postnatal care in low- and middle-income countries: a systematic review and meta-analysis. Bull World Health Organ.

[9] Dhaher E, Mikolajczyk RT, Maxwell AE, Krämer A (2008). Factors associated with lack of postnatal care among Palestinian women: A cross-sectional study of three clinics in the West Bank. BMC Pregnancy Childbirth.

[10] World Health Organization. WHO recommendations on postnatal care of the mother and newborn. World Health Organization. 2014. 24624481

[11] Clapp MA, Little SE, Zheng J, Robinson JN (2016). A multi-state analysis of postpartum readmissions in the United States. Am J Obstet Gynecol.

[12] Nam JY, Park E-C (2020). The relationship between severe maternal morbidity and a risk of postpartum readmission among Korean women: a nationwide population-based cohort study. BMC Pregnancy Childbirth.

[13] Kanyighe C, Channon A, Tadesse E, Madise N, Changole J, Bakuwa E, Malunga E, Stones RW (2008). Determinants of post-partum maternal mortality at Queen Elizabeth Central Hospital, Blantyre, Malawi: a case-control study 2001–2002. Afr J Reprod Health.

[14] Hurt LS, Alam N, Dieltiens G, Aktar N, Ronsmans C (2008). Duration and magnitude of mortality after pregnancy in rural Bangladesh. Int J Epidemiol.

[15] Moher D, Liberati A, Tetzlaff J, Altman DG, Group TP (2009). Preferred Reporting Items for Systematic Reviews and Meta-Analyses: The PRISMA Statement. PLOS Med.

[16] Veritas Health Innovation Covidence systematic review software. In: Covidence. http://www.covidence.org/. Accessed 9 Jan 2021

[17] UNDP (2018). 2018 Statistical Update: Human Development Indices and Indicators.

[18] World Health Organization. WHO technical consultation on postpartum and postnatal care. World Health Organization. 2010. 26269861

[19] Downs SH, Black N (1998). The feasibility of creating a checklist for the assessment of the methodological quality both of randomised and non-randomised studies of health care interventions. J Epidemiol Community Health.

[20] Bebell LM, Ngonzi J, Siedner MJ, Muyindike WR, Bwana BM, Riley LE, Boum Y, Bangsberg DR, Bassett IV (2018). HIV Infection and risk of postpartum infection, complications and mortality in rural Uganda. AIDS Care.

[21] Harrison MS, Pasha O, Saleem S (2017). A prospective study of maternal, fetal and neonatal outcomes in the setting of cesarean section in low- and middle-income countries. Acta Obstet Gynecol Scand.

[22] Ngonzi J, Bebell LM, Fajardo Y (2018). Incidence of postpartum infection, outcomes and associated risk factors at Mbarara regional referral hospital in Uganda. BMC Pregnancy Childbirth.

[23] Oladapo OT, Lamina MA, Sule-Odu AO (2007). Maternal morbidity and mortality associated with elective Caesarean delivery at a university hospital in Nigeria. Aust N Z J Obstet Gynaecol.

[24] Igbaruma S, Olagbuji B, Aderoba A, Kubeyinje W, Ande B, Imarengiaye C (2016). Severe maternal morbidity in a general intensive care unit in Nigeria: clinical profiles and outcomes. Int J Obstet Anesth.

[25] Ojengbede OA, Morhason-Bello IO, Galadanci H, Meyer C, Nsima D, Camlin C, Butrick E, Miller S (2011). Assessing the role of the non-pneumatic anti-shock garment in reducing mortality from postpartum hemorrhage in Nigeria. Gynecol Obstet Invest.

[26] Yego F, D’Este C, Byles J, Williams JS, Nyongesa P (2014). Risk factors for maternal mortality in a Tertiary Hospital in Kenya: a case control study. BMC Pregnancy Childbirth.

[27] Bauserman M, Lokangaka A, Thorsten V (2015). Risk factors for maternal death and trends in maternal mortality in low- and middle-income countries: a prospective longitudinal cohort analysis. Reprod Health.

[28] Smaill FM, Grivell RM (2014). Antibiotic prophylaxis versus no prophylaxis for preventing infection after cesarean section. Cochrane Database Syst Rev.

[29] Pasha O, Saleem S, Ali S (2015). Maternal and newborn outcomes in Pakistan compared to other low and middle income countries in the Global Network’s Maternal Newborn Health Registry: an active, community-based, pregnancy surveillance mechanism. Reprod Health.

[30] Ozumba BC, Anya SE (2002). Maternal deaths associated with cesarean section in Enugu, Nigeria. Int J Gynaecol Obstet.

[31] Suwal A, Shrivastava VR, Giri A (2013). Maternal and fetal outcome in elective versus emergency cesarean section. JNMA J Nepal Med Assoc.

[32] Robson MS (2001). Classification of caesarean sections. Fetal Matern Med Rev.

[33] Nemetchek B, English L, Kissoon N, Ansermino JM, Moschovis PP, Kabakyenga J, Fowler-Kerry S, Kumbakumba E, Wiens MO (2018). Paediatric postdischarge mortality in developing countries: a systematic review. BMJ Open.

[34] Garenne M, Mbaye K, Bah MD, Correa P (1997). Risk factors for maternal mortality: a case-control study in Dakar hospitals (Senegal). Afr J Reprod Health.

[35] Aminu M, Unkels R, Mdegela M, Utz B, Adaji S, van den Broek N (2014). Causes of and factors associated with stillbirth in low- and middle-income countries: a systematic literature review. BJOG.

[36] Di Mario S, Say L, Lincetto O (2007). Risk factors for stillbirth in developing countries: a systematic review of the literature. Sex Transm Dis.

[37] McClure EM, Saleem S, Pasha O, Goldenberg RL (2009). Stillbirth in developing countries: a review of causes, risk factors and prevention strategies. J Matern Fetal Neonatal Med.

[38] Uganda Bureau of Statistics - UBOS and ICF (2018). Uganda Demographic and Health Survey 2016.

[39] Wiens MO, Kissoon N, Kabakyenga J (2018). Smart Hospital Discharges to Address a Neglected Epidemic in Sepsis in Low- and Middle-Income Countries. JAMA Pediatr.

[40] Wiens MO, Kumbakumba E, Larson CP (2015). Postdischarge mortality in children with acute infectious diseases: derivation of postdischarge mortality prediction models. BMJ Open.

[41] Liang LD, Kotadia N, English L, Kissoon N, Ansermino JM, Kabakyenga J, Lavoie PM, Wiens MO (2018). Predictors of Mortality in Neonates and Infants Hospitalized With Sepsis or Serious Infections in Developing Countries: A Systematic Review. Front Pediatr.

