# Supplementary material for: Risk factors for postpartum maternal mortality and hospital readmission in low- and middle-income countries: a systematic review
Source: BMC Pregnancy Childbirth. 2023 Apr 29;23:303. doi: 10.1186/s12884-023-05459-y (PMC10148415; doi:10.1186/s12884-023-05459-y)
Supplement: Supplementary file 2 — Additional file 2. EMBASE search strategy (database inception – January 9, 2021). [file 12884_2023_5459_MOESM2_ESM.doc]

Additional file 2: EMBASE search strategy (database inception – January 9, 2021)

| **#** | **Search** |
| --- | --- |
| **1** | puerperium/ |
| **2** | (postpartum or puerperal or postnatal or post-natal or post-partum).mp |
| **3** | (post adj2 delivery).mp |
| **4** | (following adj2 birth).mp |
| **5** | 1 or 2 or 3 or 4 |
| **6** | maternal mortality/ |
| **7** | maternal death/ |
| **8** | (maternal adj2 death).mp |
| **9** | (maternal adj2 mortalit*).mp |
| **10** | 6 or 7 or 8 or 9 |
| **11** | readmission or re-admission |
| **12** | exp hospital readmission/ |
| **13** | 11 or 12 |
| **14** | 10 or 13 |
| **15** | 1 and 14 |
