# Supplementary material for: Risk factors for postpartum maternal mortality and hospital readmission in low- and middle-income countries: a systematic review
Source: BMC Pregnancy Childbirth. 2023 Apr 29;23:303. doi: 10.1186/s12884-023-05459-y (PMC10148415; doi:10.1186/s12884-023-05459-y)
Supplement: Supplementary file 3 — Additional file 3. CINAHL search strategy (database inception – January 9, 2021). [file 12884_2023_5459_MOESM3_ESM.doc]

Additional file 3: CINAHL search strategy (database inception – January 9, 2021)

| **#** | **Search** |
| --- | --- |
| **1** | (MH “Postpartum Period+”) OR (MM “Puerperium”) |
| **2** | postpartum or puerperal or postnatal or post-natal or post-partum |
| **3** | post N2 delivery |
| **4** | following N2 birth |
| **5** | 1 or 2 or 3 or 4 |
| **6** | (MH “Maternal Mortality”) |
| **7** | Maternal Death |
| **8** | maternal N2 death |
| **9** | maternal N2 mortalit* |
| **10** | 6 or 7 or 8 or 9 |
| **11** | (MH “Readmission”) |
| **12** | readmission or re-admission |
| **13** | 11 or 12 |
| **14** | 10 or 13 |
| **15** | 5 and 14 |
