# Supplementary material for: Risk factors for postpartum maternal mortality and hospital readmission in low- and middle-income countries: a systematic review
Source: BMC Pregnancy Childbirth. 2023 Apr 29;23:303. doi: 10.1186/s12884-023-05459-y (PMC10148415; doi:10.1186/s12884-023-05459-y)
Supplement: Supplementary file 4 — Additional file 4. Downs and Black checklist for the assessment of the methodological quality of both randomized and non-randomized studies. [file 12884_2023_5459_MOESM4_ESM.doc]

Additional file 4: Downs and Black checklist for the assessment of the methodological quality of both randomized and non-randomized studies

| **Criteria** | **Study Score**  ***(Yes = 1***  ***No = 0)*** | | | | | | |
| --- | --- | --- | --- | --- | --- | --- | --- |
|  | **Bebell [20]** | **Harrison [21]** | **Kanyinghe [13]** | **Ngonzi [22]** | **Oladapo [23]** | **Igbaruma [24]** | **Ojengbede [25]** |
| *Is the hypothesis/aim/objective of the study clearly described?* | 1 | 1 | 1 | 1 | 1 | 1 | 1 |
| *Are the main outcomes to be measured clearly described in the Introduction or Methods section?* | 1 | 1 | 1 | 1 | 1 | 1 | 1 |
| *Are the characteristics of the patients included in the study clearly described?* | 1 | 1 | 1 | 1 | 1 | 1 | 1 |
| *Are the interventions of interest clearly described?* | 1 | 1 | 1 | 1 | 1 | 1 | 1 |
| *Are the distributions of principal confounders in each group of subjects to be compared clearly described?* | 1 | 1 | 0 | 1 | 0 | 0 | 0 |
| *Are the main findings of the study clearly described?* | 1 | 0 | 1 | 1 | 1 | 1 | 1 |
| *Does the study provide estimates of the random variability in the data for the main outcomes?* | 1 | 1 | 1 | 1 | 1 | 1 | 1 |
| *Have the characteristics of patients lost to follow-up been described?* | 1 | 0 | 0 | 0 | 0 | 0 | 0 |
| *Have actual probability values* *been reported (e.g. 0.035 rather than <0.05) for the main outcomes except where the probability value is less than 0.001?* | 1 | 0 | 1 | 0 | 1 | 1 | 1 |
| *Were the subjects asked to participate in the study representative of the entire population from which they were recruited?* | 1 | 1 | 1 | 1 | 0 | 1 | 1 |
| *Were those subjects who were prepared to participate representative of the entire population from which they were recruited?* | 1 | 1 | 1 | 1 | 0 | 0 | 1 |
| *Were the staff, places, and facilities where the patients were treated, representative of the treatment the majority of patients receive?* | 1 | 1 | 1 | 1 | 1 | 1 | 1 |
| *Was an attempt made to blind study subjects to the intervention they have received?* | 0 | 1 | 0 | 0 | 0 | 0 | 0 |
| *Was an attempt made to blind those measuring the main outcomes of the intervention?* | 0 | 1 | 0 | 0 | 0 | 0 | 0 |
| *If any of the results of the study were based on “data dredging”, was this made clear?* | 1 | 1 | 1 | 1 | 1 | 1 | 1 |
| *In trials and cohort studies, do the analyses adjust for different lengths of follow-up of patients, or in case-control studies, is the time period between the intervention and outcome the same for cases and controls?* | 1 | 1 | 0 | 1 | 0 | 0 | 0 |
| *Were the statistical tests used to assess the main outcomes appropriate?* | 1 | 1 | 1 | 1 | 1 | 1 | 1 |
| *Was compliance with the intervention/s reliable?* | 1 | 1 | 1 | 1 | 1 | 1 | 1 |
| *Were the main outcome measures used accurate (valid and reliable)?* | 1 | 1 | 1 | 1 | 1 | 1 | 1 |
| *Were the patients in different intervention groups (trials and cohort studies) or were the cases and controls (case-control studies) recruited from the same population?* For example, patients for all comparison groups should be selected from the same hospital. The question should be answered unable to determine for cohort and case-control studies where there is no information concerning the source of patients included in the study. | 1 | 1 | 1 | 1 | 1 | 1 | 1 |
| *Were study subjects in different intervention groups (trials and cohort studies) or were the cases and controls (case-control studies) recruited over the same period of time?* | 1 | 1 | 1 | 1 | 1 | 1 | 1 |
| *Were study subjects randomised to intervention groups?* | 0 | 0 | 0 | 0 | 0 | 0 | 0 |
| *Was the randomised intervention assignment concealed from both patients and health care staff until recruitment was complete and irrevocable?* | 0 | 0 | 0 | 0 | 0 | 0 | 0 |
| *Was there adequate adjustment for confounding in the analyses from which the main findings were drawn?* | 1 | 1 | 0 | 1 | 0 | 0 | 0 |
| *Were losses of patients to follow-up taken into account?* | 1 | 0 | 0 | 1 | 0 | 0 | 0 |
| *Did the study have sufficient power to detect a clinically important effect where the probability value for a difference being due to chance is less than 5%?* | 1 | 0 | 0 | 1 | 0 | 0 | 0 |
| **Total Score** | **22** | **19** | **16** | **20** | **14** | **15** | **16** |
